# Pressure Distribution during Negative Pressure Wound Therapy of Experimental Abdominal Compartment Syndrome in a Porcine Model

**DOI:** 10.3390/s18030897

**Published:** 2018-03-17

**Authors:** Adrienn Csiszkó, Klaudia Balog, Zoltán Attila Godó, Gyula Juhász, Katalin Pető, Ádám Deák, Mariann Berhés, Norbert Németh, Zsolt Bodnár, Zsolt Szentkereszty

**Affiliations:** 1 Institute of Surgery, Faculty of Medicine, University of Debrecen, H-4032 Debrecen, Egyetem tér 1, Hungary; csiszko@gmail.com (A.C.); klaudia.balog@live.com (K.B.); szentkerzs@gmail.com (Z.S.); 2 Department of Information Technology, Faculty of Informatics, University of Debrecen, H-4032 Debrecen, Egyetem tér 1, Hungary; magortaltos@gmail.com (Z.A.G.); gyuszi32@gmail.com (G.J.); 3 Department of Operative Techniques and Surgical Research, Faculty of Medicine, University of Debrecen, H-4032 Debrecen, Egyetem tér 1, Hungary; kpeto@med.unideb.hu (K.P.); deak.adam@med.unideb.hu (Á.D.); nemeth@med.unideb.hu (N.N.); 4 Department of Anaesthesiology and Intensive Care, Faculty of Medicine, University of Debrecen, H-4032 Debrecen, Egyetem tér 1, Hungary; bermarjan@yahoo.co.uk; 5 Department of Surgery, Letterkenny University Hospital, F92VX8D Letterkenny, Ireland

**Keywords:** intra-abdominal pressure, abdominal compartment syndrome, pressure sensor, negative pressure wound therapy, open abdomen

## Abstract

(1) Introduction: Negative pressure wound therapy (NPWT) is a frequently applied open abdomen (OA) treatment. There are only a few experimental data supporting this method and describing the optimal settings and pressure distribution in the abdominal cavity during this procedure. The aim of our study was to evaluate pressure values at different points in the abdominal cavity during NPWT in experimental abdominal compartment syndrome (ACS) animal model; (2) Methods: In this study (permission Nr. 13/2014/UDCAW), 27 Hungahib pigs (15.4–20.2 kg) were operated on. ACS was generated by implanting a plastic bag in the abdomen through mini-laparotomy and filled with 2100–3300 mL saline solution (37 °C) to an intraabdominal pressure (IAP) of 30 mmHg. After 3 h, NPWT (Vivano Med^®^ Abdominal Kit, Paul Hartmann AG, Germany) or a Bogota bag was applied. The NPWT group was divided into −50, −100 and −150 mmHg suction groups. Pressure distribution to the abdominal cavity was monitored at 6 different points of the abdomen via a multichannel pressure monitoring system; (3) Results: The absolute pressure levels were significantly higher above than below the protective layer. The values of the pressure were similar in the midline and laterally. Amongst the bowels, the pressure values changed periodically between 0 and −12 mmHg which might be caused by peristaltic movements; (4) Conclusions: The porcine model of the present study seems to be well applicable for investigating ACS and NPWT. It was possible to provide valuable information for clinicians. The pressure was well distributed by the protective layer to the lateral parts of the abdomen and this phenomenon did not change considerably during the therapy.

## 1. Introduction

Intra-abdominal hypertension (IAH) and abdominal compartment syndrome (ACS) are common findings in critically ill patients. Although diagnostic and therapeutic guidelines were described by The Abdominal Compartment Society (WSACS), controversy still surrounds the treatment. The treatment of IAH/ACS is basically conservative and semiconservative (minimal invasive intervention). Surgical intervention is required when these treatment options fail. The basic surgical approach is open abdomen (OA) therapy [1,2]. 

In understanding the underlying pathophysiologic events, several experimental animal models have been used. Small animal models are useful for investigating pathophysiologic changes. Large animal models are particularly advantageous for evaluating surgical techniques and their effects. The most commonly used large animal model is the porcine model [3].

Negative pressure wound therapy (NPWT) is an increasingly applied method of open abdomen therapy in clinical conditions, although there is limited experimental data supporting its effectiveness and describing its possible adverse effects [4,5,6]. 

The authors used a porcine model for experimental abdominal compartment syndrome and OA therapy, emphasizing NPWT and describing its optimal settings. Abdominal compartment syndrome was induced in pigs and pressure distribution within the abdominal cavity was measured during negative pressure wound therapy. 

## 2. Materials and Methods

This study was performed at the Department of Operative Techniques and Surgical Research, Faculty of Medicine, University of Debrecen, Hungary. It was approved by the local institutional committee on animal research (permission No. 13/2014/UDCAW). 

Twenty-six Hungahib pigs (15.4–20.2 kg) were kept in standard conditions (22–23 °C) before the experiment. The operations were performed under general anaesthesia by intramuscular administration of 15 mg/bwkg ketamine and 1 mg/bwkg xylazine. The animals were ventilated through tracheostomy with assisted air and oxygen ventilation. A central venous catheter was placed in the left jugular vein. The left femoral artery was used as the arterial access point. A urinary catheter was introduced suprapubically to measure urine output. 

After inducing anaesthesia, a plastic bag was implanted in the abdomen through mini-laparotomy (30 mm midline incision) and filled with 2100–3300 mL pre-heated (37 °C) saline solution until an intra-abdominal pressure (IAP) of 30 mmHg was reached. The abdominal wall was temporarily closed in an airtight manner by two layers of running sutures. The balloon was also used for monitoring the IAP. Body temperature, urinary output, haemodynamic parameters including heart rate, peripheral oxygen saturation, blood pressure, mean arterial pressure (MAP), central venous pressure (CVP) were monitored throughout the experiment. 

After 3 h, the bag was deflated and removed through a total midline laparotomy and the open abdomen therapy was initiated. The abdomen was temporarily closed by Bogota bag and a urinary catheter was inserted with sutures to the wound edges in six animals (untreated pigs from historic data). In case of the other 20 pigs NPWT (Vivano Med^®^ Abdominal Kit, PAUL HARTMANN AG, Hamburg, Germany) was applied. A micro-structured organ protection layer was placed on the small intestines under the parietal peritoneum, which was covered by a foam dressing and a self-adhesive layer on the top. The set was then connected to a special device (VivanoTec^®^ Unit, PAUL HARTMANN AG, Germany) to create reduced pressure within the abdominal cavity. 

**Figure 1:**
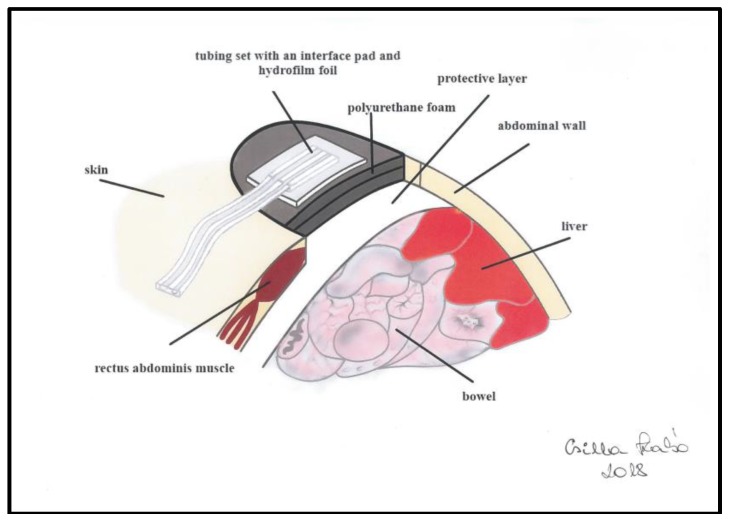
The negative pressure wound therapy system in the open abdomen management. In the hermetically closed system the continuous suction is transmitted to the abdominal cavity by the application of a special polyurethane foam. The bowels are covered by a protective layer to avoid direct contact between the abdominal organs and the foam. The negative pressure is provided by an electric device.

The principle of NPWT is the constant transmission of negative pressure to the open abdomen. In the hermetically closed system the continuous suction transmission to the abdominal cavity is provided by the special application of polyurethane foam. The bowels are covered by a protective layer to avoid the direct contact between the abdominal organs and the polyurethane foam. The negative pressure is provided by an electric device (Figure 1).

**Figure 2:**
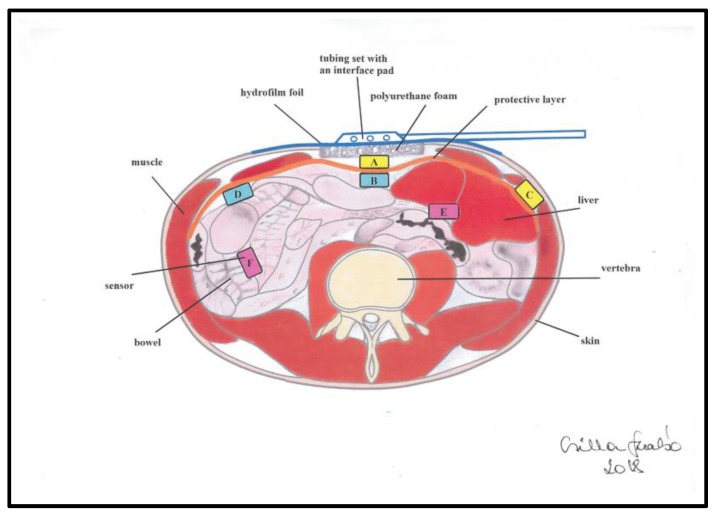
Schematic image of the strategic positioning of six pressure sensors in the abdominal cavity used for intra-abdominal pressure measurement in an experimental animal model. A: midline, above the layer; B: midline, under the layer; C: laterally, above the layer; D: laterally, under the layer; E: midline, among the bowels; F: laterally, among the bowels.

The NPWT group was divided into three subgroups. In seven animals, −50 mmHg suction was applied; another seven pigs were included in the −100 mmHg group and six in the −150 mmHg group. During the experiment design process, we encountered difficulties in the monitoring of intra-abdominal pressure in different levels of the abdominal cavity. The microcontroller based multichannel pressure sensor system (MBMPSS), designed by our working group solved this issue. It is able to measure relative pressure levels at six different points of the abdomen. The pressure measurement provided by this system was referenced to atmospheric pressure. Each sensor was covered by a special foam protecting the bowel wall from direct contact. Two sensors were placed laterally (8 cm from the lateral side of the wound edge), another two in the midline above and under the protective layer. Finally, two further sensors were placed between the small bowels (Figure 2). These six sensor points had specific clinical significance in our experiment. We wanted to get information about how pressure generated by the device is conducted to the lateral and deeper regions of the abdomen, to observe the effectiveness of this NPWT system. The device measured pressure every 10 s throughout the negative pressure treatment interval of 120-min (720 data/each sensor). 

Results are expressed as mean ± SD. Each measurement time points in the presented tables and diagrams represents the sum of 10 measured values in every 5 min of each animal from the beginning (0 point) of NPWT. The relationship between the summary of the measured sensors (50 measurements of each sensor at each pressure level) was analysed using the correlation analysis method. The assessment of the correlation analysis is summarized in Table 1.

## 3. Results

**Figure 3:**
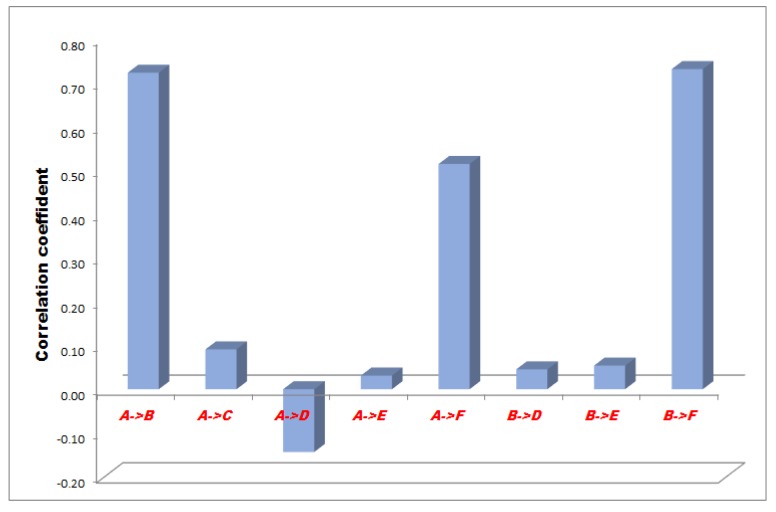
Results of statistical (correlation) analysis between the different sensor pairs during Negative pressure wound therapy (NPWT) applying at 50 mmHg suction. Sensors represent exact measurement points in the abdominal cavity. A: midline, above the layer; B: midline, under the layer; C: laterally, above the layer; D: laterally, under the layer; E: midline, among the bowels; F: laterally, among the bowels.

**Figure 4:**
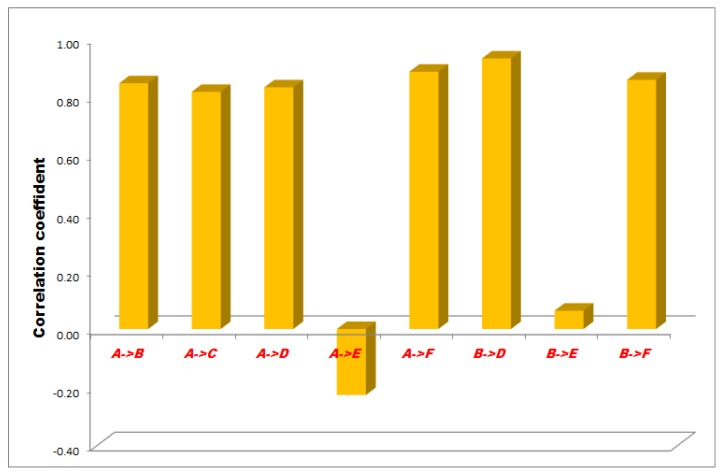
Results of statistical (correlation) analysis between the different sensor pairs during NPWT applying at 100 mmHg suction. Sensors represent exact measurement points in the abdominal cavity. A: midline, above the layer; B: midline, under the layer; C: laterally, above the layer; D: laterally, under the layer; E: midline, among the bowels; F: laterally, among the bowels.

**Figure 5:**
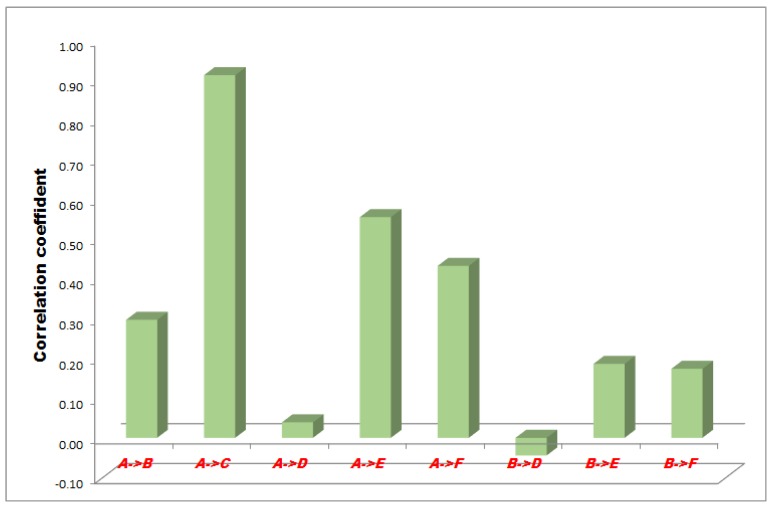
Results of statistical (correlation) analysis between the different sensor pairs during NPWT applying at 150 mmHg suction. Sensors represent exact measurement points in the abdominal cavity. A: midline, above the layer; B: midline, under the layer; C: laterally, above the layer; D: laterally, under the layer; E: midline, among the bowels; F: laterally, among the bowels.

The correlation between the measurement points of minus 50, 100 and 150 mmHg NPWT are represented in Figure 3, Figure 4 and Figure 5.

In the −50 mmHg NPWT group, between the A and F sensors the correlation was a moderate and significant relationship, between the A and B, and B and F sensors the correlation was high with a strong relationship and between the A and C, A and E, B and D, B and E sensors the correlation was slight, almost negligible. Between the A and D sensors there was no linear relationship (Figure 3).

In the −100 mmHg NPWT group, between the B-E sensors the correlation was low with an irrelevant relationship, between the A and B, A and C, A and D, A and F and B and F sensors the correlation was high with a strong relationship and between the B and D sensors the correlation was very high with a strong dependable relationship. There was no linear relationship between the A and E sensors (Figure 4).

In the −150 mmHg NPWT group, between the A and D sensors the correlation was low with an irrelevant relationship, between the A and E and A and F sensors the correlation was moderate with a significant relationship, between A and C the correlation was very high with a strong dependable relationship. A low correlation but a definite relationship has been proved between the A and B, B and E, B and F sensors. Between B and D there was no linear relationship (Figure 5).

**Figure 6:**
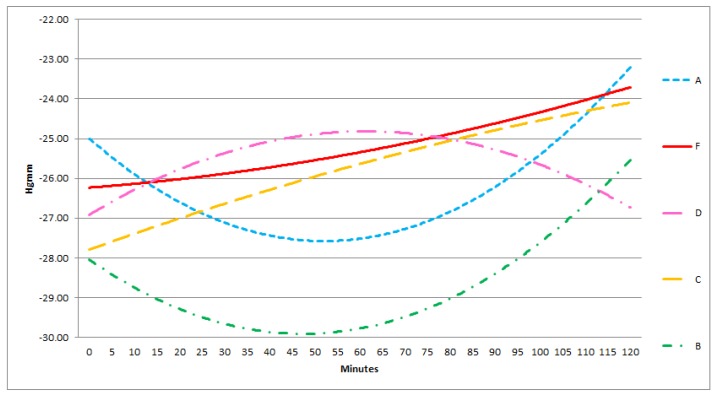
Results of statistical (correlation) analysis between the different sensor pairs during NPWT applying at 50 mmHg suction. The pressure values and their changes in time was measured by different sensors at −50 mmHg NPWT. Sensors represent exact measurement points in the abdominal cavity. (The pressures at sensors A–F are presented enlarged also. A: midline, above the layer; B: midline, under the layer; C: laterally, above the layer; D: laterally, under the layer; E: midline, among the bowels; F: laterally, among the bowels.)

**Figure 7:**
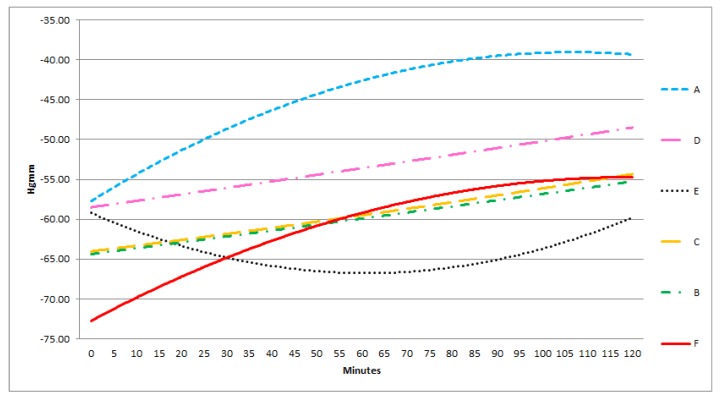
Results of statistical (correlation) analysis between the different sensor pairs during NPWT applying at 100 mmHg suction. The pressure values and their changes in time was measured by different sensors at 100 mmHg NPWT. Sensors represent exact measurement points in the abdominal cavity. (The pressures at sensors A–F are presented enlarged also. A: midline, above the layer; B: midline, under the layer; C: laterally, above the layer; D: laterally, under the layer; E: midline, among the bowels; F: laterally, among the bowels.)

**Figure 8:**
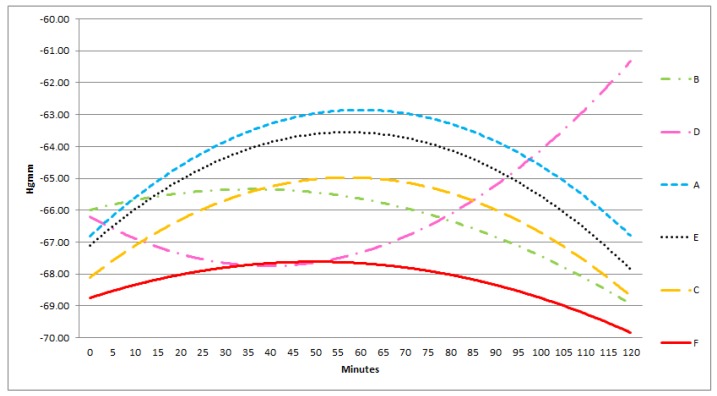
Results of statistical (correlation) analysis between the different sensor pairs during NPWT applying at 150 mmHg suction. The pressure values and their changes in time was measured by different sensors at 150 mmHg NPWT. Sensors represent exact measurement points in the abdominal cavity. (The pressures at sensors A–F are presented enlarged also. A: midline, above the layer; B: midline, under the layer; C: laterally, above the layer; D: laterally, under the layer; E: midline, among the bowels; F: laterally, among the bowels.)

In each group, there was a good correlation between the sensors in the middle and the lateral positions, in contrast with the poor correlation between the superficially and deeply positioned sensors. Furthermore, the absolute pressure in all three groups amongst the bowels was significantly lower than below or above the protective layer. In the lateral and medial positions the pressure levels were approximately the same (Figure 6, Figure 7 and Figure 8).

During NPWT the measured pressure levels showed no significant difference in time. (Figure 6, Figure 7 and Figure 8).

At −50 mmHg pressure level, A and B are convex, D is a concave curve, C and F are linear functions, E is a monotonically decreasing curve. Monotonity of functions A, B, D changes after 50–60 min from the 0 point of the experiment.

At −100 mmHg, monotonity of curve A is unchanged during the experiment. A and F are concave. Function E changes into a concave curve. B is a linear, D is an apparently linear function.

At −150 mmHg pressure level, A, C, E, F, are convex and they change in monotonity after 60 min. B is a concave, D is a convex curve.

Monotonity of the curves are the most equalized at −100 mmHg.

## 4. Discussion

ACS is defined as a sustained elevation of IAP above 20 mmHg (with or without abdominal perfusion pressure (APP) <60 mmHg) that is associated with a new organ dysfunction/failure [1]. ACS is a life-threatening condition and leaving it untreated leads to multi-organ failure and death. When IAP is consistently over 20 mmHg despite conservative treatment and organ dysfunction is present, surgical intervention is mandated. The aim of the surgical intervention is to control bleeding, prevent contamination of the abdominal cavity and to decompress it.

There are many temporary abdominal closure (TAC) techniques such as skin only sutures, the application of absorbable meshes, Bogota bag, zipper systems, Wittmann Patch and the different NPWT methods. Open abdomen (OA) complications are well-known and these include entero-atmospheric fistula formation, fluid and protein loss, catabolism, adhesions in the abdominal cavity, fascial retraction among many others [1,2,3,7,8]. According to the WSACS guidelines, NPWT is recommended in the treatment of ACS as it provides superior results to simple packing techniques [1]. NPWT is now increasingly being used in clinical scenarios, although there is limited experimental data demonstrating its effects and complications. There is a lack of evidence-based recommendations regarding to the optimal settings for NPWT.

It is extensively published that large animals especially pigs are suitable for investigating the effect of ACS on hemodynamics, organ function, circulation and evaluate conservative and surgical treatment options, due to their anatomy and pathophysiology being relatively close to humans [9,10,11,12,13,14]. Before starting the study design Andrew Kirkpatrick’s excellent experimental work on pigs was extensively studied among many others [15]. As there has been no previous paper of physiologic changes related to laparoscopic surgery (artificial intra-abdominal hypertension) in weightlessness, Kirkpatrick studied anesthetized pigs in parabolic flight providing important data for future laparoscopic surgical procedures performed in space on human beings. The available literature widely supports that the results of an experimental porcine model are well/best applicable to human conditions. ACS definition in an animal model can be stated if an artificially increased IAP leads to circulatory, renal or respiratory insufficiency [3]. In our pig model 3 h of IAH (30 mmHg) was enough for the above-mentioned consequences of ACS to appear. 

Every former animal ACS model showed that well-timed decompression reduces mortality, has favourable effect on the ACS induced changes on hemodynamics and organ function [16,17]. NPWT was found superior to other TAC methods by authors investigating the different TAC techniques [4,18]. However, Benninger et al. advised to use the bag or zipper system first and to use NPWT only a few days later in order to prevent reoccurrence of IAH/ACS [19]. 

There is limited data in the literature with regard to the pressure distribution in the abdominal cavity during NPWT. The present study demonstrates the pressure distribution in lateral regions and between the bowels. The clinical significance of these results is important for understanding the pressure distribution in the abdominal cavity and to design further methods for optimal pressure control during NPWT. We tried to find out the ideal positions for the pressure sensors, as well as the most effective and equilibrated pressure level. It is known from the clinical experience that during the OA management (acute necrotizing pancreatitis, faecal peritonitis, intra-abdominal sepsis, polytrauma patient), it is crucial for maintaining a stable and satisfactory level of negative pressure in the deep layers of the peritoneal cavity. The AbThera^®^ system is an excellent option to provide these requirements in the superficial and lateral parts of the abdominal cavity; however, the central zone still remains a hidden part. The idea to design a system for pressure measurement provided for every part of the peritoneal cavity rooted in this. In this study three negative pressure set up were assessed: the 50 mmHg, the 100 mmHg and the 150 mmHg. The 100 mm Hg level seemed to be ideal because in almost every parameter investigated by us the best results were experienced at applying the NPWT at 100 mmHg suction. At this pressure level the suction effect seemed to be well equilibrated and strong enough in each part of the abdominal cavity.

There is limited data in the literature with regard to the pressure distribution in the abdominal cavity during NPWT. The present study demonstrates the pressure distribution in lateral regions and between the bowels. The clinical significance of these results is important for understanding the pressure distribution in the abdominal cavity and to design further methods for optimal pressure control during NPWT. We tried to find out the ideal positions for the pressure sensors, as well as the most effective and equilibrated pressure level. It is known from the clinical experience that during the OA management (acute necrotizing pancreatitis, faecal peritonitis, intra-abdominal sepsis, polytrauma patient), it is crucial for maintaining a stable and satisfactory level of negative pressure in the deep layers of the peritoneal cavity. The AbThera^®^ system is an excellent option to provide these requirements in the superficial and lateral parts of the abdominal cavity; however, the central zone still remains a hidden part. The idea to design a system for pressure measurement provided for every part of the peritoneal cavity rooted in this. In this study three negative pressure set up were assessed: the 50 mmHg, the 100 mmHg and the 150 mmHg. The 100 mm Hg level seemed to be ideal because in almost every parameter investigated by us the best results were experienced at applying the NPWT at 100 mmHg suction. At this pressure level the suction effect seemed to be well equilibrated and strong enough in each part of the abdominal cavity.

According to the available clinical and experimental data, NPWT increases tissue perfusion, collagen production, granulation and angiogenesis, which helps wound healing. It provides medial traction preventing lateralization of the abdominal wall or dehiscence. The fascial closure rates are estimated around 35%–92%. It ensures effective drainage in order to prevent abscess formation [5,6,8,18,20]. The Vivano Med Abdominal Kit consists of three layers. The first layer is a perforated polyethylene sheet (interface layer) for covering the abdominal organs that reduces adhesions, protects the bowels and lowers fistula rate. Second layer is a polyurethane foam to be placed within the wound edges, in order to provide medial traction and to help fluid drainage. Third layer is an adhesive film, which closes the wound airtight on the surface, it prevents fluid and heat loss and external contamination. The system is connected to a vacuum source using special tubing set. The complication rate is lower however there is minimal available experimental data on optimal settings and the ideal advisable pressure level still remains unknown. During the study, we tried to develop the ideal sensor positions to provide efficient pressure monitoring and to identify the ideal pressure level. The most challenging zone of the peritoneal cavity for sensor placement is the central part which requires not only a special “bowel friendly” sensor placement method but requires a special device too, which does not cause injuries and/or fistula formation. The microcontroller based multichannel pressure sensor system (MBMPSS), designed by us solved this issue. It is able to measure relative pressure levels at six different points of the abdomen. These six sensor points had crucial clinical significance in our experiment. We wanted to know how pressure is conducted to the lateral and deeper regions of the abdomen; concerning the possible bowel wall injury and fistula formation. We wanted to know the differences between the measurement points and their changes in time to observe the effectiveness of NPWT therapy.

It is hypothesized that the negative pressure conducted to the small bowels may elevate the risk of fistula formation, decreases tissue microcirculation and causes ischemia [4,21]. Clinical data show that the risk of fistula formation is around 0–15% [7,8]. The effect of NPWT on bowel surface has hardly been investigated and according to limited literature data NPWT may reduce small bowel wall blood flow, especially close to the visceral protective layer [21,22]. This complication might be more severe with the negative pressure applied [22]. Bjarnason et al. investigated the pressure distribution of NPWT in the abdomen and has provided data in which the foam conducts 75% of applied pressure to the abdomen [6]. The negative pressure is significantly reduced on the bowel surface but lowering the applied pressure does not further reduce pressure on the bowels [4,6]. In our model, irrespective of the suction applied (−50, −100, −150 mmHg) there were significantly lower values of negative pressure that can be measured between the small bowels in the deep layer compared to near the protective sheet. Under the protective sheet the negative pressure values are significantly lower than above it.

Bjarnason et al. has found that outside the inner layer negative pressure diminishes [6]. This is similar to our data. Pressures were similar in the middle and in the lateral regions of the abdomen, supporting the theory that the visceral layer provides excellent pressure distribution.

The negative pressure is conducted well to the area underneath the protective sheet in the midline and to the superficial lateral region of the abdominal cavity. The pressure distributed with good efficacy to the inter-intestinal space in the midline and laterally also. These findings were most pronounced with application of 100 mmHg negative pressure.

## 5. Conclusions

Pressure distribution at different points of the abdomen could be evaluated during NPWT, which helps clinicians to choose the optimal settings. The organ protection layer provides excellent pressure distribution in the abdominal cavity. The extensive cover of the abdominal organs with this layer is highly advised. The favourable effect of the Vivano Med Abdominal Kit has been proven in this animal model.

## Figures and Tables

**Table 1:** Correlation coefficient (Guilford, 1950).

Correlation Coefficient	Level of Correlation
0	no linear relationship
0–0.2 (−0.2–0)	negligible correlation with irrelevant relationship
0.2–0.4 (−0.4–−0.2)	low correlation, definite but small relationship
0.4–0.7 (−0.7–−0.4)	moderate correlation with significant relationship
0.7–0.9 (−0.7–−0.9)	high correlation with strong relationship
0.9–1 (−1–−0.9)	very high correlation with strong dependable relationship

## Abbreviations

NPWT	negative pressure wound therapy
OA	open abdomen
ACS	abdominal compartment syndrome
IAP	intra-abdominal pressure
IAH	intra-abdominal hypertension
bwkg	body weight kilogram
MAP	mean arterial pressure
CVP	central venous pressure
MBMPSS	microcontroller based multichannel pressure sensor system
SD	standard deviation
WSACS	The Abdominal Compartment Society
APP	abdominal perfusion pressure
TAC	temporary abdominal closure
